# The Impact of Maternal Supplementation of Fish Oil and/or Probiotics During Pregnancy on the Serum Metabolomic Profile From Infancy to Childhood: Secondary Analysis of a Randomized Placebo-Controlled Trial

**DOI:** 10.1016/j.cdnut.2025.107553

**Published:** 2025-09-09

**Authors:** Veera Houttu, Dattatray Mongad, Noora Houttu, Lotta Saros, Chunpeng Zhang, Jenni Viitaharju, Tero Vahlberg, Leo Lahti, Kirsi Laitinen

**Affiliations:** 1 Nutrition and Food Research Center, Faculty of Medicine, University of Turku, Turku, Finland; 2 Department of Computing, Faculty of Technology, University of Turku, Turku, Finland; 3 Integrative Physiology and Pharmacology Unit, Institute of Biomedicine, Faculty of Medicine, University of Turku, Finland; 4 Department of Biostatistics, University of Turku and Turku University Hospital, Turku, Finland; 5 Department of Obstetrics and Gynecology, Turku University Hospital, Wellbeing Services County of Southwest Finland, Turku, Finland

**Keywords:** childhood, infancy, metabolomics, pregnancy, fish oil, probiotics, RCT, longitudinal

## Abstract

**Background:**

Supplementation with probiotics and fish oil may modify circulating serum metabolites, but the extent to which their impacts can be transferred from mother to child is unknown.

**Objectives:**

To investigate the impact of perinatal exposure to fish oil and/or probiotics on serum metabolomic profile in early childhood.

**Methods:**

Children (*n* = 300) of pregnant females receiving fish oil+placebo, probiotics+placebo, fish oil+probiotics, or placebo+placebo [fish oil: 1.9-g docosahexaenoic acid and 0.2-g eicosapentaenoic acid; probiotics: *Lacticaseibacillus rhamnosus* HN001 and *Bifidobacterium animalis* subsp. *lactis* 420] from early pregnancy to 6-mo postpartum were observed until 5–6 y of age. Serum metabolomic profiles were analyzed using nuclear magnetic resonance metabolomics. The intervention’s impact on the overall metabolomic profile was assessed using permutation analysis of variance with multi-omics factor analysis utilized to infer latent factors that capture main sources of variability within each group, followed by a univariate comparison between the intervention groups at each age. The time effect was analyzed using a mixed model.

**Results:**

We observed significant differences in the concentrations of fatty acids and lipoproteins at 6 months across the intervention groups (false discovery rate < 0.05). The main effects included higher serum concentration of docosahexaenoic acid and n–3 fatty acids, a higher ratio of n–3/n–6 fatty acids in fish oil+placebo and fish oil+probiotics groups compared with placebo+placebo, along with higher concentrations of lipids and cholesterol derivatives in very large high-density lipoproteins. At 6 mo, there were no significant differences in these metabolites for the probiotics+placebo group compared to placebo+placebo. At 1, 2, and 5–6 y, a total of 24 metabolites were affected by the intervention (non-significant after multiple correction).

**Conclusions:**

Maternal intervention with fish oil alone and in combination with probiotics induces alterations in the metabolic profile at 6 mo of age, as demonstrated by increased circulating n–3 fatty acids and lipids in high-density lipoproteins.

**Clinical Trial Registry number:**

NCT01922791 (https://clinicaltrials.gov/ct2/show/NCT01922791.

## Introduction

A child’s metabolism, including nutrient utilization and energy and lipid metabolism, regulates her/his growth and development, and also influences the risk of diseases, extending up to adulthood [[1], [2], [3]]. It is noteworthy that these regulatory processes may be subject to influence as early as the intrauterine stage of development [4]. Therefore, disruptions in maternal metabolic health during pregnancy, such as may occur with overweight and obesity, may be of importance and have detrimental effects on the fetus’s metabolic regulation [5]. In this regard, it has been claimed that the perinatal stage serves as an ideal time window to modify maternal exposures such that they will induce beneficial metabolic changes in the fetus, and subsequently promote the child’s development and future health [6].

As we have previously reported, a supplementation of combined fish oil and probiotics during pregnancy induced alterations in circulating compounds related to lipid, glucose, and amino acid metabolism [6], as well as in the composition of the gut microbiota in pregnant females with overweight or obesity [7]. Both fish oil and probiotics possess beneficial properties, that is, fish oil, which is rich in n–3 long-chain polyunsaturated fatty acids (n–3 LC-PUFA), in particular DHA and EPA, is known to alleviate metabolic perturbations, like insulin resistance and cardiovascular outcomes [8,9] by reducing inflammation and improving lipid metabolism [10] as well as being beneficial to the development of the fetal visual functions and central-nervous system [11]. Administration of probiotics, such as Bifidobacterium and Lactobacillus, has also been shown to influence lipid homeostasis via regulation of the gut microbiota and inflammation [12]. The effects of these supplements can potentially be enhanced by their combination. For example, the administration of n–3 LC-PUFA with probiotics caused a more profound effect on the lipid content of lipoproteins of mothers with overweight and obesity than either supplement alone, as we have previously shown [6].

Importantly, although the maternal intake of fish oil and probiotics during pregnancy did not influence the prevalence of gestational diabetes mellitus (GDM) [13], it may evoke cross-generational effects, as evidenced by a reduced risk for overweight and obesity and recurrent wheezing in their 2-year-old children [14,15]. Here, high-throughput metabolomics was utilized to investigate the links between *in utero* dietary exposures and health implications on children. This approach has made it possible to pinpoint specific metabolic processes and fluxes in the first years of life in childhood [16]. In a previous study, maternal supplementation of n–3 LC-PUFA from mid-pregnancy to 1-wk postpartum reduced the levels of metabolites related to fatty acids (n–6 LC-PUFAs, monounsaturated and saturated fatty acids) and tryptophan, whereas there were higher levels of metabolites related to tyrosine and glutamic acid in children at the age of 4–8 mo [17]. Thus far, the research on the effects of perinatal supplementation of n–3 LC-PUFA on the offspring’s metabolic profile is scarce, and no studies exist on the combined administration of n–3 LC-PUFA and probiotics in a longitudinal setting from pregnancy until 6 y of age.

Although the impact of maternal health and nutrition on the child’s later health is acknowledged, the metabolic processes, for instance, lipid and amino acid metabolism, in early childhood induced by specific nutritional exposure during pregnancy, have remained unexplored. Furthermore, although the use of metabolomic approaches in the context of pediatrics is making progress, longitudinal dietary studies from early infancy to later childhood are limited, and none have involved mother–child pairs with the focus on perinatal and postnatal fish oil and probiotic exposure. Metabolic processes like those captured by targeted metabolomics, by profiling lipid metabolites in early life, may serve as potential mechanisms linking maternal fish oil and/or probiotics intervention to various conditions. Therefore, we aimed to investigate the influence of maternal supplementation of fish oil or/and probiotics during pregnancy and 6-mo postpartum on the metabolomic profile of children as they grew from 6 mo to 5–6 y of age.

## Methods

### Study design and subjects

This study utilizes data from a double-blinded, randomized, placebo-controlled, single-center intervention with fish oil and/or probiotic supplementation (ClinicalTrials.gov: NCT01922791) for which females were recruited in Southwest Finland between October 2013 and July 2017. The original trial design has been described previously in detail, and the primary outcomes were to investigate whether fish oil and/or probiotics supplements decrease the risk of GDM in females and allergies in children [13]. Briefly, a total of 439 pregnant females were enrolled in the study and randomly assigned to the intervention. Pregnant females (gestational wk < 18) were eligible with BMI (in kg/m^2^) ≥ 25, and who did not present with multifetal pregnancy, GDM diagnosis, and metabolic or inflammatory diseases. In this study, our aim was to investigate the influence of maternal fish oil and/or probiotics supplementation on the metabolomic profile of their children ≤5–6 y of age, which is a predefined secondary outcome of the original trial.

Demographics (age, education, smoking habits) were collected by interviewing the participants who filled in questionnaires during the first study visit in early pregnancy. Information on delivery was obtained from hospital medical records. Prepregnancy BMI was determined by dividing self-reported prepregnancy weight obtained from health clinic records by height measured with a wall stadiometer to the nearest 0.1 cm at the first study visit.

**FIGURE 1:**
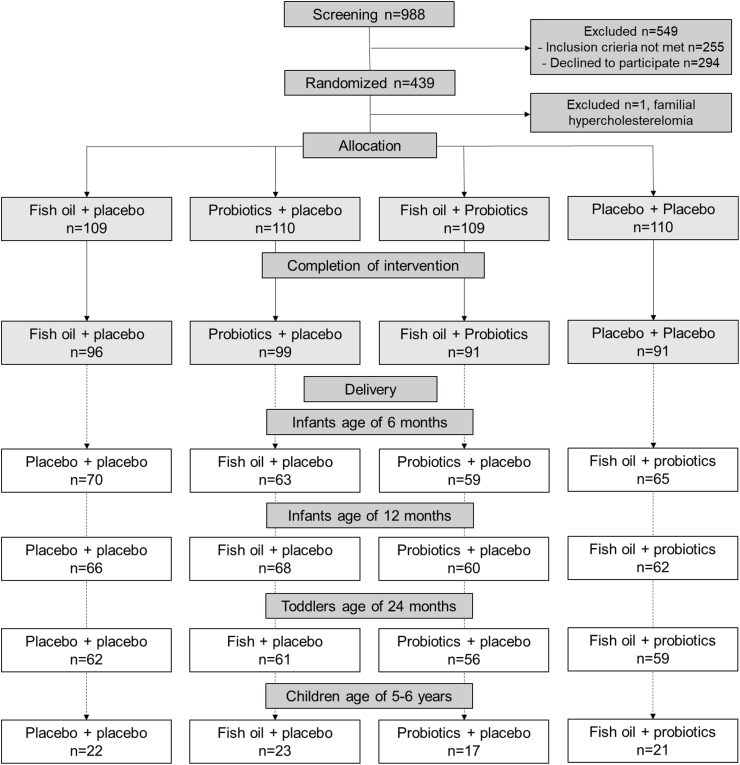
Flowchart of the present study of children with available serum samples for nuclear magnetic resonance determined metabolomics analysis.

The children of the participating females were observed ≤5–6 y of age. In this present study, at least one blood sample was available from a total of 300 children at any timepoint for metabolomics analysis; 257 children at 6 mo, 256 at 1 y, 238 at 2 y, and 83 at 5–6 y of age (Figure 1). Information regarding the child’s health and growth measurements was obtained from hospital and pediatric health clinic medical records. Children’s birth and growth data consisting of weight-for-length percentage and weight-for-height percentage, weight for age SD scores, as well as weight, height, and head circumference, were determined as previously described [15]. The duration of breastfeeding and the use of infant formula (until the age of 2 y) were assessed during the follow-up visits orally and via questionnaires. The mothers filled in 3-d food diaries in early pregnancy. The study followed the guidelines of the Declaration of Helsinki, and the Ethics Committee of the Hospital District of Southwest Finland gave prior approval for the study protocol before the study commenced. All mothers and 5–6 y old children provided written informed consent.

### Intervention

The females were randomly assigned in a double-blind manner into 4 groups: *1)* fish oil+placebo; *2)* probiotics+placebo; *3)* fish oil+probiotics; and *4)* placebo+placebo at the first study visit (13.9 ± 2.1 gestational wk). The allocation was conducted according to the females’ parity and history of GDM. The fish oil capsules contained 2.4 g of n–3 LC-PUFA (1.9-g DHA, 0.22-g EPA, and 0.28-g other n–3 fatty acids, for example, docosapentaenoic acid; Croda Europe Ltd) and the probiotic capsules contained 10^10^ colony-forming units of *Lacticaseibacillus* (formerly *Lactobacillus) rhamnosus* HN001 (ATCC SD5675; DuPont, Niebüll, Germany) and *Bifidobacterium animalis* subsp. lactis 420 (DSM 22089; DuPont) per capsule. The placebo for fish oil contained an equal amount of medium-chain fatty acids as the fish oil capsules, and the placebo for the probiotics contained microcrystalline cellulose with an identical shape, size, and color as the active capsule. Females were instructed to consume 2 fish oil and 1 probiotic capsule throughout the intervention from the first study visit until 6 mo postpartum. The intervention with fish oil and/or probiotics during pregnancy was both safe and well tolerated [13]. The overall study compliance was 88.4% based on the returned fish oil capsules, that is, a mean of 91.8% ± 15.9% of the consumed capsules [13]. This was further confirmed when nuclear magnetic resonance (NMR) determined the serum lipids of the mothers, guaranteeing their fish oil intake [6].

### Blood sampling and metabolomics analysis

Blood samples of the children at the age of 6 mo, 1 y, and 2 y were withdrawn from antecubital vein after a varying time of eating [1.50 h (IQR) 1.00, 2.00] at 6 mo, *n* = 225; 1.50 h (IQR: 1.00, 2.00) at 1 y, *n* = 227; 2.00 h (1.50, 2.50) at 2 y, *n* = 87], and after an overnight fast at the age of 5–6 y of age [10.5 h (2.57, 7.71), *n* = 49] by a certified nurse with experience in pediatrics. The serum was separated and stored in aliquots at −80^0^C until the analysis. The serum metabolites were determined using a high-throughput NMR metabolomics platform (Nightingale Health Ltd) as previously described [18]. The platform consists of a targeted set of 250 metabolite biomarkers of lipid and glucose metabolism, amino acids, ketone bodies, and glycoprotein acetyls. The bioinformatic analyses were carried out separately for 173 metabolites analyzed as concentrations and ratios of n–6/n–3 fatty acids, and for 77 metabolites analyzed as the ratio of total lipids [the sum of phospholipids, cholesterol esters, free cholesterol (FC), and triglycerides] or total fatty acids (the sum of fatty acids). The missing values in metabolite data were imputed using the *impute_metabimpute_gsimp*() function from the *imputomics* R package (v0.1.3).

### Statistical and bioinformatics analysis

The statistical analysis for the clinical characteristics in the 4 diet intervention groups was carried out in R (v4.4.2) [19]. The normality of distribution of the continuous data was visually observed from histograms and statistically evaluated by the Shapiro-Wilk test. Normally distributed data were described as means with standard deviations, skewed data as medians with IQRs, and categorical data as frequencies with percentages. Statistical differences were tested to evaluate global differences in diet intervention. ANOVA was used for normally distributed continuous variables and Kruskal-Wallis for skewed variables. Categorical variables were compared using Pearson's chi-square or Fisher’s exact test. A *P* value of < 0.05 was considered statistically significant for clinical outcomes.

The study was originally powered according to the primary outcome of GDM incidence and allergy in the children [13]. In the present study, the metabolomic profile was a predefined secondary outcome. The power for the effect of the intervention on the child’s metabolomics analysis could not be calculated because during the time of intervention, the application of a metabolomics approach to study human metabolism was only just emerging, and no prior data on the effects of maternal supplementation of fish oil and/or probiotics on child serum metabolomics existed. Nevertheless, the number of study participants was considered sufficient to detect differences in serum metabolites in response to the intervention, as reported previously in mothers [6,20].

The dissimilarities in the metabolomic profile were assessed with miaViz (v1.16.0) [21]. The data was ln_10_-transformed using a pseudocount (equal to the minimum nonzero value), then z-score normalized across features. Principal component analysis (PCA) was performed on transformed data, and global group differences were evaluated using Permutational Analysis of Variance (PERMANOVA) statistical comparison. Multi-omics factor analysis 2 (MOFA2) multigroup framework analysis (v1.16.0) [22] was used to identify the sources of variability in the metabolomic profile within each intervention group, to characterize shared patterns across groups, and to identify which are specific to each individual group. To test for differentially abundant metabolites between intervention groups, 2 complementary methods were used. First, using DAtest R package (v2.8.0), we applied the nonparametric approach [23]. Then, the Kruskal-Wallis test with Benjamini-Hochberg method to control for the false discovery rate (FDR) for multiple comparisons was applied to evaluate the global differences between the intervention groups, followed by post hoc pairwise comparisons using Mann-Whitney U-test with Bonferroni correction and Benjamini-Hochberg method. An FDR < 0.05 was considered statistically significant. Both Bonferroni and FDR-adjusted *P* values are reported. A heatmap of pairwise significant metabolites was generated using ln_2_-fold change values, which were normalized by median absolute deviation. Second, we used MaAsLin2 (v1.19.0) [24] to identify the associations between intervention groups and the children's metabolic profile with log-transformation and total sum scaling normalization. The model was adjusted with confounding factors, maternal prepregnancy BMI, and smoking during pregnancy because these variables differed between the intervention groups at the age of 6 mo (see Supplemental Table 1). The child’s sex was also considered in the model as sex is known to influence the metabolomic profile in early childhood [25,26]. To examine the effect of diet intervention across age, we used a generalized linear mixed model with a gamma distribution and log link function. The model included the fixed effects for the interaction between diet intervention group and age, and a random intercept for each subject. ”*Placebo+placebo*” was the reference level for the intervention group. The fixed-effect structure was aligned with covariates used in the MaAsLin2 analysis, which includes maternal prepregnancy BMI, smoking during pregnancy, and child sex. All visualizations were created using ggplot2 (v3.5.1) [27], ggpubr (v0.60) [28], and patchwork (v1.3.0) [29].

## Results

### Maternal and child characteristics

The characteristics of the mothers and birth data of the children in relation to the intervention are presented in Table 1. More than half of the mothers were living with overweight (62.0%, 25 ≤ BMI < 30), whereas the rest were living with obesity (38.0%, BMI ≥ 30). Most of the births were via vaginal delivery (84.0%), and half of the children were girls (50.0%). With respect to the intervention groups, smoking during pregnancy differed between the intervention groups (*P* < 0.002), being highest in the placebo+placebo group (26.0%). Further characteristics of the children at the age of 6 months, and further at 1 year, 2 and 5–6 years of age in relation to the intervention are presented in the Supplemental Table 1.

**TABLE 1:** Characteristics of mothers and children according to the intervention groups.

	Total *n*	All	Group *n*	Fish oil + placebo	Probiotics + placebo	Fish oil + probiotics	Placebo + placebo	*P* value^1^
Mother baseline:								
Mother's age (y)	300	30.6 (4.9)	78/71/74/77	30.6 (4.9)	31.1. (4.2)	31.0 (4.6)	30.5 (4.2)	0.8
Education (college or university education) (*n*, %)^1^	300	194 (65.0)	78/71/74/77	54 (69.0)	47 (66.0)	45 (61.0)	48 (62.0)	0.7
Primipara (*n*, %)^1^	300	154 (51.0)	78/71/74/77	43 (55.0)	37 (52.0)	36 (49.0)	38 (49.0)	0.8
Smoking before pregnancy (*n*, %)^1^	300	50 (17.0)	78/71/74/77	7 (9.0)	17 (24.0)	6 (8.1)	20 (26.0)	0.002
Prepregnancy BMI (kg/m^2^)^2^	300	28.6 (26.4, 31.8)	78/71/74/77	29.4 (27.3, 32.9)	27.8 (26.4, 30.3)	28.3 (25.8, 31.4)	29.0 (26.4, 31.7)	0.12
Obesity and overweight (n (%))	300		78/71/74/77					0.2
with overweight (25 ≤ BMI < 30)^1^		185 (62.0)		41 (53.0)	49 (69.0)	47 (64.0)	48 (62.0)	
with obesity (BMI ≥ 30)^1^		115 (38.0)		37 (47.0)	22 (31.0)	27 (36.0)	29 (38.0)	
Diet intake								
Energy (KJ)	297	8.1 (7.0, 9.5)	76/69/73/75	7.7 (6.8,8.7)	8.3 (7.2, 9.5)	8.3 (7.0, 10.0)	8.2 (7.2, 9.5)	0.11
Protein (E%)	297	16.6 (14.6, 18.6)	76/69/73/75	16.2 (14.5, 18.1)	16.7 (14.0, 18.4)	16.4 (14.8, 18.3)	17.1 (14.6, 19.2)	0.6
Carbohydrate (E%)	297	46 (6)	76/69/73/75	46 (7)	46 (7)	46 (6)	45 (6)	0.6
Total fat (E%)	297	35 (6)	76/69/73/75	35 (7)	35 (6)	34 (6)	35 (7)	0.8
PUFA (E%)	297	5.4 (4.6, 6.4)	76/69/73/75	5.6 (4.7, 6.7)	5.3 (4.4, 6.6)	5.2 (4.4, 5.9)	5.5 (4.7, 6.3)	0.4
MUFA (E%)	297	12.1 (10.2, 13.7)	76/69/73/75	12.3 (10.5, 13.9)	11.9 (10.3, 13.4)	11.5 (10.2, 13.0)	12.3 (10.2, 13.9)	0.4
SFA (E%)	297	12.8 (3.0)	76/69/73/75	12.6 (3.4)	12.7 (2.7)	12.8 (2.7)	13.0 (3.1)	0.9
Fiber (g)	297	17.7 (13.6, 22.2)	76/69/73/75	16.7 (13.3, 21.3)	16.4 (13.4, 21.6)	17.5 (13.7, 20.8)	19.4 (15.8, 23.3)	0.2
Mother pregnancy:								
Smoking during pregnancy (*n*, %)^1^	298	9 (3.0)	78/70/73/77	0 (0.0)	1 (1.4)	3 (4.1)	5 (6.5)	0.09
GDM diagnosis (*n*, %)^1^	294	25 (32.0)	77/71/71/75	25 (32.0)	21 (30.0)	19 (27.0)	21 (28)	0.9
Gestational weeks at the delivery (wk)^2^	300	39.9 (39.0, 40.6)	78/71/74/77	39.9 (39.3, 40.4)	40.1 (39.0, 40.7)	39.9 (39.0, 40.7)	39.7 (38.6, 40.6)	0.7
Vaginal delivery (*n*, %)^1^	300	251 (84.0)	78/71/74/77	65 (83.0)	59 (83.0)	62 (84.0)	66 (86.0)	>0.9
Child birth:								
Girl sex (*n*, %)^1^	299	149 (50.0)	77/71/74/77	40 (52.0)	33 (46.0)	42 (57.0)	34 (44.0)	0.4
Born preterm (< 37+0 gw) (*n*, %)^1^	300	17 (5.7)	78/71/74/77	4 (5.1)	4 (5.6)	7 (9.5)	2 (2.6)	0.3
Birth weight (g)^2^	300	3620 (3295, 3975)	78/71/74/77	3633 (3325, 3920)	3610 (3275, 4005)	3620 (3300, 3980)	3600 (3290, 3990)	>0.9
Birth height (cm)^2^	195	51.0 (49.0, 52.0)	76/71/71/77	51.0 (50.0, 51.3)	51.0 (49.5, 52.0)	51.0 (49.0, 52.0)	51.0 (49.0, 52.0)	>0.9
Birth head circumference (cm)^1^	196	35.0 (34.0, 36.5)	76/71/70/77	35.3 (34.5, 36.0)	35.5 (34.0, 3.5)	35.0 (34.0, 36.0)	35.0 (34.0, 36.0)	0.9

E%, energy percent; GDM, gestational diabetes mellitus; gw, gestational weeks.

1 One-way ANOVA for normally distributed continuous variables; Kruskal-Wallis rank sum test for nonnormally distributed continuous variables; Pearson's Chi-squared test or Fisher's exact test for categorical variables.

2 Categorical data expressed as frequency (%); continuous nonnormally distributed data expressed as median (IQR), otherwise continuous normally distributed data expressed as mean (standard deviation).

### The impact of maternal supplementation with fish oil and/or probiotics on the global child serum metabolomic profile at different ages

**FIGURE 2:**
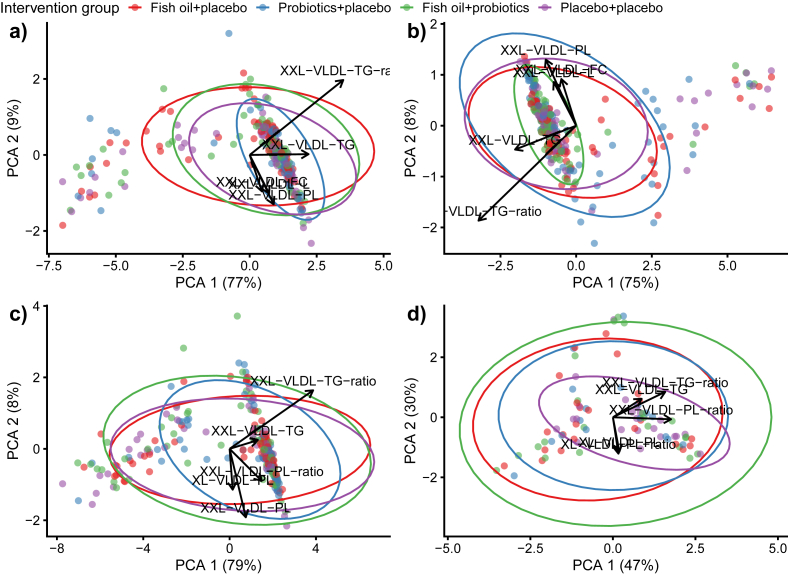
Principal component analysis (PCA) plot of the metabolites of children at 6 months of age (a), 1 year of age (b), 2 years of age (c) and 5–6 years of age (d) according to the intervention groups. Metabolite data are log10 transformed followed by Z-transformation with Euclidean distance. Large VLDL, L-VLDL; very large VLDL, XL-VLDL; extremely large VLDL, XXL-VLDL; free cholesterol, FC; phospholipids, PL; triacylglycerols, TG; to total lipids ratio, ratio.

No statistically significant separation (PERMANOVA) between dietary intervention groups at any of the examined ages, 6 mo (*P* = 0.11), 1 y (*P* = 0.66), 2 y (*P* = 0.26), and 5–6 y (*P* = 0.87), was visible in PCA; Figure 2). This indicates that the global metabolomic differences between groups were subtle and could not be shown in the unsupervised PCA visualization (PCA loadings are presented in Supplemental Figure 1).

**FIGURE 3:**
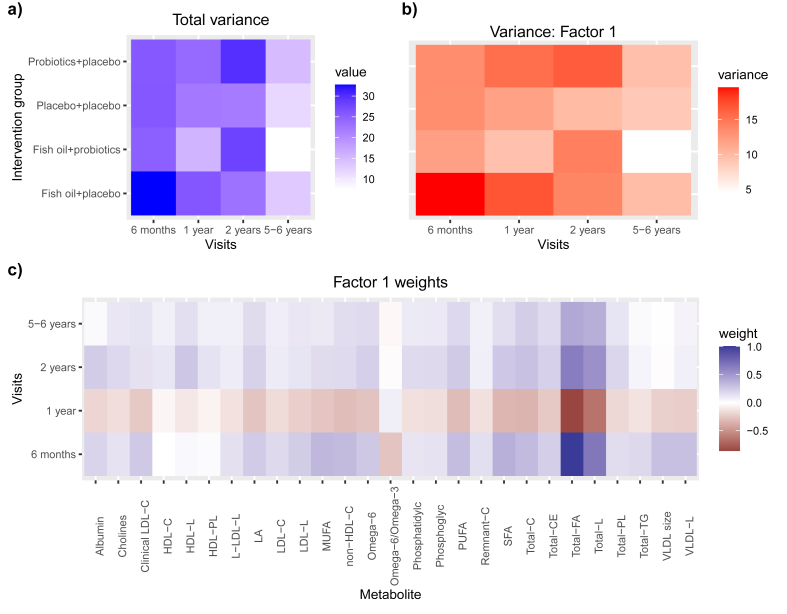
Multi-omics factor analysis 2 multigroup framework analysis explaining the variability (A) in each intervention group at 6 mo, 1 y, 2 y, and 5–6 y of ages. The figure shows the total explained variance of concentrations of metabolites (A), the explained variance of concentrations of metabolites in factor 1 (B) and the weights of associated top 10 concentrations of metabolites per visit explaining variability in factor 1 (C).

To further explore age-specific patterns in metabolite-driven variation, MOFA2 was applied using a multigroup framework (concentrations and ratios separately, and dietary intervention groups were considered as groups and age groups as views). With respect to the concentrations, at the age of 6 mo and 1 y, fish oil+placebo group exhibited the highest variance (33% and 26%, respectively), whereas at 2 y and 5–6 y, the probiotics+placebo group exhibited the highest variance (30% and 15%, respectively) (Supplemental Table 2, Figure 3A). A declining trend in total variance was observed with increasing age, with the lowest variance noted in the children aged 5–6 y (8%–15%), and with the lowest variance being in the placebo+placebo group. Furthermore, factor 1 captured the largest proportion of variance across samples, as expected from the multi-omics factor analysis (MOFA) model, which orders the latent factors according to a decreasing explanatory power. The factor 1 variance of concentrations of metabolites was highest in the fish oil+placebo group at the age of 6 mo (20%), 1 y (17%), and 5–6 y (9%), whereas in the probiotics+placebo group, it occurred at 2 y (17%) (Supplemental Table 2, Figure 3B). In all ages, the factor 1 variance was driven primarily by total fatty acids (Supplemental Table 2, Figure 3C).

With respect to ratios, when we examined the total variance at 6 months and 1 year of age, the fish oil+placebo group exhibited the highest variance (93% at both ages), whereas at 2 and 5–6 y, the placebo+placebo group displayed the highest variance (93% and 56%, respectively) (Supplemental Figure 2A, Supplemental Table 3). The highest factor 1 variance was evident in the fish oil+probiotics group at age 6 mo (56%), in the placebo+placebo group at the age of 1 y (59%), 2 y (54%), and 5–6 y (38%) (Supplemental Figure 2B, Supplemental Table 3). At 6 mo, 1 y, and 2 y, the majority of factor 1 variance was driven by the ratio of triacylglycerols to total lipids in extremely large VLDL, whereas at 5–6 y of age, it was attributable to the ratio of cholesterol to total lipids in extremely large VLDL (Supplemental Figure 2C, Supplemental Table 3). Overall, ratios of metabolites related to VLDL lipoproteins emerged as the primary contributor to the variation across all age groups.

### The impact of maternal intervention on serum metabolomics in 6-month-old children

**FIGURE 4:**
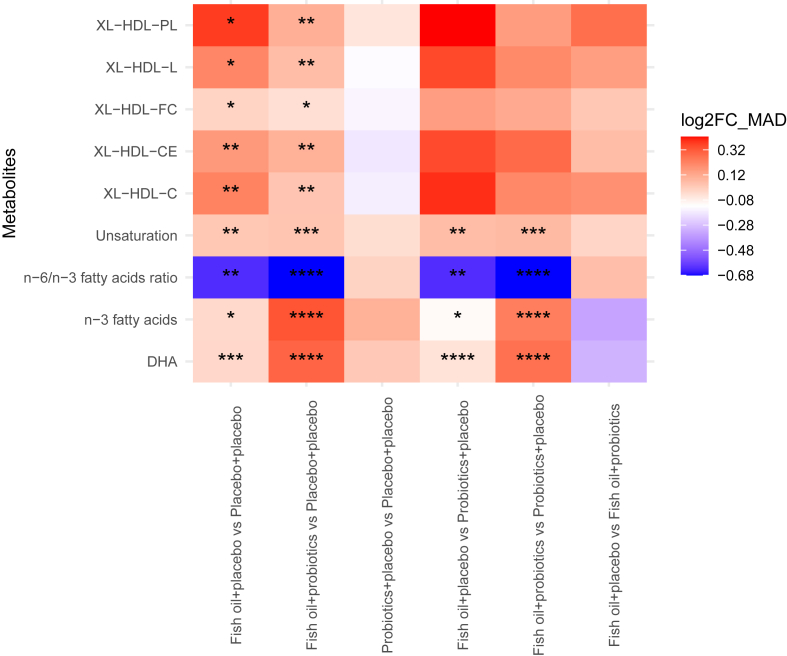
Heatmap of the effect size (normalized ln_2_-fold by median absolute deviation) of significantly changed concentration of serum metabolites of 6-month-old children in comparison between the intervention groups, maternal intake of fish oil+placebo (*n* = 63), fish oil+probiotics (*n* = 65) probiotics+placebo (*n* = 59), and placebo+placebo (*n* = 70) supplementation. ∗ FDR > 0.05 ∗∗ FDR > 0.001 ∗∗∗ FDR > 0.0001 ∗∗∗∗ FDR > 0.00001. DHA, docosahexaenoic acid; XL-HDL-C, cholesterol in very large high-density lipoprotein; XL-HDL-CE, cholesteryl esters in very large high-density lipoprotein; XL-HDL-FC, free cholesterol in very large high-density lipoprotein; XL-HDL-L, total lipids in very large high-density lipoprotein; XL-HDL-PL, phospholipids in very large high-density lipoprotein.

At the age of 6 mo, the maternal dietary intervention was associated with significant changes in the concentrations of 25 metabolites, as identified using the Kruskal-Wallis multigroup test (*P* < 0.05). Of these, 9 metabolites remained statistically significant after correction for multiple testing (Kruskal-Wallis, FDR < 0.05; Supplemental Table 4). Namely, the concentrations of fatty acids, DHA, and n–3 fatty acids (the sum of DHA, EPA, and α-linolenic acid), and the degree of unsaturation of fatty acids (the number of carbon-carbon double bonds in the fatty acid carbon chain) were higher, whereas the ratio of n–6 to n–3 fatty acids was lower in both the fish oil+placebo and the fish oil+probiotics groups than that of the placebo+placebo and probiotics+placebo groups (Mann-Whitney with Bonferroni correction and Benjamini-Hochberg method, FDR < 0.05; Figure 4, Supplemental Table 5). With respect to the lipoproteins, the composition of very large HDL, namely, cholesterol, cholesteryl esters (CEs), FC, phospholipids (PL), and total lipids in the particles was significantly higher in the fish oil+placebo and fish oil+probiotics groups in comparison to placebo+placebo (Mann-Whitney with Bonferroni correction and Benjamini-Hochberg method, FDR < 0.05; Figure 4, Supplemental Table 5). These metabolites, but not PL in very large HDL, were also higher in the children of the participants in the fish oil+placebo and fish oil+probiotics groups than those whose mothers had received probiotics+placebo (Mann-Whitney with Bonferroni correction and Benjamini-Hochberg method, FDR < 0.05; Figure 4, Supplemental Table 5). Nonetheless, total lipids were not affected by the consumption of fish oil+placebo when compared to probiotics+placebo. There were no significant differences in the comparison of fish oil+placebo to fish oil+probiotics, nor of probiotics+placebo to placebo+placebo (Mann-Whitney with Bonferroni correction and Benjamini-Hochberg method, FDR < 0.05; Figure 4, Supplemental Table 5). In a multivariate model adjusted for the child’s sex, maternal prepregnancy BMI and smoking during pregnancy, the interventions involving fish oil+placebo and fish oil+probiotics were associated with an increased abundance in the concentration of DHA, and the fish oil+probiotics intervention with an elevated abundance of n–3 fatty acids, as well as very large HDL and reduced ratio of n–6/n–3 fatty acids and (MaAsLin2, FDR < 0.05) (Supplemental Table 6). Finally, the maternal consumption of probiotics+placebo did not seem to exert any impact on the serum metabolomics in the offspring.

In terms of the metabolites analyzed as total lipid and total fatty acids ratios, the maternal dietary intervention was associated with statistically significant differences in the ratios of 30 metabolites at the age of 6 mo, as identified using the Kruskal-Wallis multigroup test (*P* < 0.05). Of these, 7 metabolites, including lipid ratios related to n–3 fatty acids and the lipid composition of HDL and LDL particles, differed statistically between the groups (Kruskal-Wallis FDR < 0.05; Supplemental Table 7). Namely, in the group-wise comparisons, the following ratios were significantly changed: higher ratios were detected for n–3 fatty acids and DHA to total fatty acids; PL to total lipids in medium LDL and small LDL; and FC to total lipids in small HDL, whereas lower ratios were evident for PL to total lipids in large HDL and CE to total lipids in medium LDL in the fish oil+placebo and fish oil+probiotics when compared to the placebo+placebo group (Mann-Whitney with Bonferroni correction and Benjamini-Hochberg method, FDR < 0.05; Supplemental Table 8). The results were essentially the same in the fish oil+placebo versus probiotics+placebo comparisons, but 2 ratios, that is, PL to total lipids in small LDL and PL to total lipids in large HDL, did not differ between the groups. Similarly, the comparisons between fish oil+probiotics and probiotics+placebo group revealed that the results were essentially the same, except for the ratio of CE to total lipids in medium LDL and the PL to total lipids ratio in large HDL, which did not differ between the groups. There were no differences between probiotics+placebo as compared to placebo+placebo or fish oil+placebo when compared to fish oil+probiotics (Mann-Whitney with Bonferroni correction and Benjamini-Hochberg method, FDR < 0.05; Supplemental Table 8). In the multivariate model adjusted for covariates, increases in n–3 fatty acids and DHA to total fatty acids ratio were present in the fish oil+placebo group, as was the DHA to total fatty acids ratio in the fish oil+probiotics group (MaAsLin2, FDR < 0.05; Supplemental Table 9.

### The impact of the maternal intervention on the serum metabolomics of 1-, 2-, and 5–6-year-old children

At the age of 1 y, in the initial inspection, a total of 18 metabolites were assessed as having been influenced by the maternal intervention (Kruskal-Wallis, *P* < 0.05) (Supplemental Table 4). These metabolites included amino acids, lipoproteins, lipids, and metabolites related to energy metabolism, but none remained significant after multiple testing (Kruskal-Wallis, FDR > 0.05). At the age of 2 y, in the initial inspection, although it did seem that the creatine level had been significantly influenced by the interventions in 2-year-old children (Kruskal-Wallis, *P* < 0.05), after multiple testing correction, this difference disappeared (Kruskal-Wallis, FDR < 0.05) (Supplemental Table 4). At the age of 5–6 y, in the initial univariate statistical analysis, 5 metabolites, that is, lactate, pyruvate, alanine, triglycerides in large LDL, and intermediate density lipoprotein particles, appeared to have been influenced by the intervention, (Kruskal-Wallis, *P* < 0.05), but once again, none remained significant after correcting for multiple testing (Kruskal-Wallis, FDR < 0.05) (Supplemental Table 4). Furthermore, none of the associations were significant in the multivariate model after adjustment with prepregnancy BMI, smoking, and child sex when correcting for multiple testing in any of the age groups (MaAsLin2, FDR > 0.05) (Supplemental Table 6).

When individual metabolites were analyzed as the ratio of one metabolite to the total, then there were changes in the ratio of PL to total lipids in large LDL at the age of 1 y. Although it seemed that there were also changes with respect to 7 metabolites which had been influenced by the maternal intervention, that is, the ratio of PL, cholesterol, CE and TG to total lipids in different lipoproteins at the age of 2 y and 6 metabolites, including the ratio of PL, cholesterol, CE, FC, and TG to total lipids in different lipoproteins at the age of 5–6 (Kruskal-Wallis, *P* < 0.05), these differences were no longer statistically significant after correction for multiple testing (Kruskal-Wallis, FDR > 0.05; Supplemental Table 7). In the multivariate model analyses, none of the ratios of the metabolites to total lipids or total fatty acids were statistically significant in any of the age groups (MaAsLin2, FDR > 0.05) (Supplemental Table 9).

### The impact of maternal intervention on serum metabolites of children from 6 mo to 5–6 y of age

**FIGURE 5:**
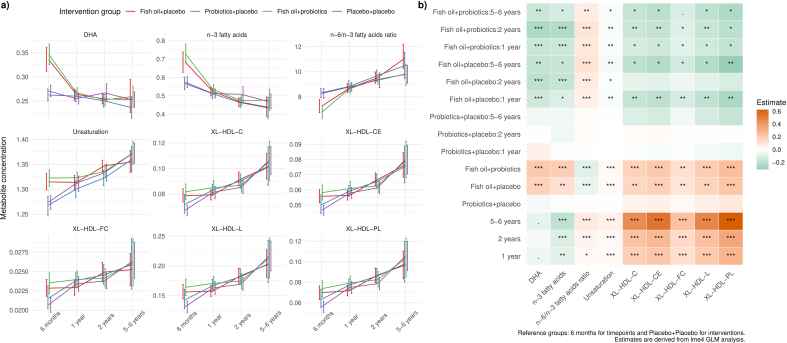
Interaction between the maternal intervention and time on the concentrations of serum metabolites of children. (A) Changes in metabolite concentrations across visits are stratified by intervention groups with means and error bars. (B) Estimates from linear mixed-effects models illustrating the main effects of maternal intervention groups and time, as well as their interactions with children's metabolic profiles.

We further investigated the change in selected metabolites (statistically significant according to Kruskal-Wallis in the multigroup testing) across the timepoints and intervention groups using linear regression models adjusted with covariates. “*Placebo+placebo*” was set as the reference level for the intervention group, positive estimates indicate higher expected metabolite levels compared with “*placebo+placebo*”; negative estimates indicate lower levels. With respect to the concentrations, overall, those of DHA and n–3 fatty acids decreased along with age, whereas the n–6/n–3 fatty acid ratio, as well as the lipids in very large HDL, increased when the children grew from being babies to young children (Figure 5). The statistically significant effects (FDR < 0.05, Figure 5 heatmap), indicated that the provision to the mothers of probiotics+placebo did not have overall (main) effect on the children’s metabolite concentrations whereas with the exception of the n−6/n−3 fatty acid ratios, metabolites increased more in the fish oil+placebo and fish oil+probiotics groups as compared to the placebo+placebo group (Figure 5 heatmap).

Similarly, when the calculation was conducted in terms of ratios, there were declines in the ratios of *1)* PL to total fatty acids in large HDL; *2)* CE to total lipids in medium LDL; *3)* n–3 fatty acids to total fatty acids; in contrast, the ratio of PL to total lipids in medium LDL increased (Supplemental Figure 3). Interestingly, the ratio of DHA to total fatty acids as well as that of FC to total lipids in small HDL decreased from 6 mo until 2 y and then fluctuated until 5–6 y. Furthermore, the ratio PL to total lipids in small LDL increased from 6 mo until 1 y and then exhibited a decreasing trend as the children aged to 5–6 y. As with the metabolites, none of the ratio showed a significant main effect of probiotics+placebo. From 6 mo to 5–6 y, the ratios of DHA, n–3 fatty acids to total fatty acids, as well as the FC-to-total lipids ratio in small HDL, decreased more in fish oil+placebo and fish oil+probiotics groups as compared to placebo+placebo (FDR < 0.05; Supplemental Figure 3, heatmap). The ratio of PL to total lipids in HDL showed an increase in fish oil+probiotics from 1 to 5–6 y and in fish oil+placebo until 2 y of age as compared to placebo+placebo. Similarly, the ratio of CE to total lipids in medium LDL increased more significantly ≤1 y of age, whereas the ratio of PL to total lipids in medium LDL showed a greater decrease during the same period compared to the placebo+placebo group. Finally, the ratio of CE in total lipids increased more until the children’s first birthday in the fish oil+probiotics group in comparison to the placebo+placebo group.

## Discussion

Our findings demonstrate that supplementing the maternal diet with fish oil and/or probiotics from early pregnancy to 6-mo postpartum influences the child’s serum lipidomic profile ≤5–6 y of age, suggesting a potential mechanism that may partially explain previous clinical findings [14,15]. The global inspection (PCA) revealed no distinct separation between the intervention groups. However, MOFA indicated that all intervention groups contributed to the variance in the metabolic profile, with this contribution decreasing over time. The detailed inspection of the differences between the groups per time-point revealed that fish oil and fish oil in combination with probiotics but not probiotics alone had influenced the metabolites at the age of 6 mo. Namely, the children of mothers who had been supplemented with fish oil alone and in combination with probiotics had higher circulating levels of n–3 fatty acids, along with a higher concentration of metabolites related to lipoprotein metabolism. This was evident with respect to the very large subfractions of HDL as defined by differences in the concentrations of different lipids (CE, FC, PL, and total lipids). Furthermore, time-interaction analyses showed that those same metabolites except the ratio of n–6/n–3 fatty acids decreased in the fish oil+placebo and fish oil+probiotics groups as compared to the placebo+placebo group from 6 mo to 5–6 y of age. In addition, the values describing the ratio of total lipids support these findings, along with revealing additional results on decreased CE and increased PL ratios with respect to total lipids in LDL particles in the participants consuming fish oil alone or in combination with probiotics.

In the present study, the maternal consumption of fish oil alone and in combination with probiotics was reflected in their offspring’s serum lipid concentrations at the age of 6 months, especially in the concentrations of n–3 fatty acids. The children were breast-fed until, on average 12-mo postpartum (see details in Supplemental Table 1). Another time for exposure is during the fetal period; this is supported by our earlier studies, where we showed that the supplementation with fish oil during pregnancy increased the maternal serum n–3 fatty acids [6,20]. It is noteworthy that the combination of fish oil and probiotics, as compared to placebo-induced effects, altered a higher number of metabolites than fish oil alone in our previous study done in pregnant females [6] than was seen in this current study conducted with children. However, when the combination of fish oil and probiotics was compared to probiotics alone, it was observed that 13 metabolites differed statistically significantly between the groups, but when the combination was compared to fish oil alone, then no differences were seen. Although the fish oil+probiotics group did not differ statistically significantly from the fish oil+placebo group, the medians of the statistically significant metabolites at 6 mo of age in fish oil+probiotics group were higher than in the fish oil+placebo group, suggesting that the probiotics may have had some synergistic effects when combined with fish oil. Similarly to evidence emerging from our previous study from the same dataset, the combination of fish oil and probiotics exerted effects on immune mediators in pregnant females, not only when compared to fish oil alone but also in comparison with probiotics alone [30]. It was shown in another study with maternal supplementation of fish oil containing 2.4-g n–3 LC-PUFA from weeks 22–26 of pregnancy, but continuing only until 1-wk postpartum, that a distinctive plasma metabolic profile could be detected in the metabolome in children at the age of 6 mo [17]. Our study extended ≤5–6 years of age, but differences in the metabolic profile were no longer detected at these later timepoints, reflecting the termination of the pregnancy intervention effect as well as the fact that to achieve stable higher concentrations of circulating serum fatty acids would require continual exposure to fish oil. This was supported by MOFA analysis, which suggests that the effects of this dietary intervention seemed to diminish with age. Interestingly, the group × time-interaction analyses showed that the metabolites that differed between fish oil or fish oil+probiotics and placebo+placebo groups at 6 mo of age, decreased or increased more until 5–6 years of age in these same groups than in placebo, an indication of longer-lasting effects. Thus, the results may be interpreted to demonstrate that early life exposure to fish oil could exert a long-lasting impact on metabolic regulation and health. This concept is supported by other studies in which early-life exposure to n–3 LC-PUFAs has had clinical benefits, including a lower risk for asthma [31]. Nonetheless, the impacts of the maternal intake of n–3 LC-PUFAs on the cardiometabolic health of the child have been contradictory. The children of mothers receiving fish oil daily during pregnancy from week 24 until 1 wk after birth presented with a higher BMI at 6 y of age [32], and later the same children seemed to have an increased risk for being overweight at 10 y of age as compared to the control group of children of mothers who consumed olive oil containing n–9 oleic acid and n–6 linoleic acid [33]. On the contrary, if the n–3 LC-PUFA supplementation was given to infants from birth to 6 mo of age, when the children reached 5 y of age, the effects were beneficial, that is, a lower waist circumference and reduced insulin resistance [34]. The variations in the results could be explained by the different composition of n–3 LC-PUFAs administered, dosage and duration of intake, the length of the follow-up, and the maternal population.

In addition to n–3 fatty acids, the concentrations of CE, FC, and PL in very large HDL particles were also increased in response to maternal consumption of fish oil alone or fish oil in combination with probiotics. We suggest that the intervention exerted beneficial effects also in this regard, as previous studies conducted in children have shown that low lipid concentrations in large and very large HDL particles are associated with an increased type 2 diabetes liability in 8-, 16-, 18-, and 25-year-old subjects [35]. This concept is supported by another study, where a small HDL particle size was associated with the presence of type 2 diabetes in children at the age of 12 y [36]. Moreover, in one study, cardiorespiratory fitness was positively associated with the concentrations of large and extra-large HDL particles in 6–8-year-old children [37]. When the amount of PL in the HDL particles was assessed, it was found that this correlated with an increased risk for cardiometabolic problems in 18-year-old female adolescents [38]. In addition to the findings related to the HDL particles, we found effects in the LDL particles when this was measured as a ratio to total lipids in the children who were 6 mo of age; there were higher ratios of PL to total lipids in medium LDL and small LDL. In contrast, the ratio of CE to total lipids in medium LDL was lower when the children’s mothers had been in either the fish oil+placebo or the fish oil+probiotics group rather than the placebo+placebo group. Since an increased level of CE in LDL is a well-known risk factor for cardiovascular diseases [39], the findings related to the lower CE to total lipids ratio in medium LDL after maternal consumption of either fish oil+placebo or fish oil+probiotics can be considered as beneficial. Similarly, the finding of a higher PL to total lipids ratio in medium and small LDL is in line with the above beneficial effect, that is, when the ratio of PL to total lipids increases, the ratio of other major components, that is, CE and TG, will decrease, both of which are known to exert detrimental effects on the body’s metabolism. Regarding the effects of the probiotics, our findings from the group-wise comparisons per age in years and with a longitudinal inspection are in line with our previous findings [6,20], as the probiotics did not influence the metabolites in the children. Nonetheless, probiotics may exert some effects through breast milk as supplementation of probiotics influences the levels n–3 fatty acids in breast milk, as we have previously shown [40].

Interestingly, before controlling for multiple comparisons, it appeared that exposure to maternal supplementation alone had been able to influence the concentrations of serum branched-chain amino acids (BCAA), isoleucine, leucine, and valine, as well as one aromatic amino acid, phenylalanine, in 1-year-old children. As far as we are aware, we are the first to report that maternal intake of fish oil and/or probiotics would be able to alter the serum concentrations of BCAAs in toddlers in a mother–child pair dietary intervention. Serum concentrations of BCAAs have been previously suggested to act as an indicator of hyperinsulinemia [41], and the concentrations of BCAAs in cord blood have been associated with adiposity in children [42]. In particular, the leucine level in the newborn has been shown to predict overweight later in childhood [43]. Thus, it could be argued that BCAAs could serve as a marker for metabolic processes and disease risks, rather than having a causal role in disease development. This topic could be further investigated with a study powered to investigate this outcome.

The main strength of our study lies in the randomized controlled trial design with dietary supplementation in a large mother–child-pair population, although the outcomes are secondary. In addition, this study has a long follow-up, that is, ≤5–6 y of age, with a large number of serum samples gathered from the children. Unfortunately, many children dropped out during the follow-up, especially at the age of 5–6 years. In this respect, these results could suffer from a lack of power, and a larger sample size may be required if one wishes to detect statistically significant differences at the later ages. Moreover, variations in the timing of blood sampling relative to food intake could potentially affect the results, as eating may alter metabolite levels. However, it is not feasible to impose prolonged fasting on very young children. It is also noteworthy that the mothers were living with overweight or obesity, and thus, these findings may not be applicable to children of mothers with normal weight. However, we chose to study this group of females who not only themselves but also their children are at increased risk for developing metabolic diseases, as they represent an important group in which interventions may be beneficial. Smoking before pregnancy differed between the intervention groups, which could potentially influence the results. Yet, the number of females who were smoking before pregnancy is still rather small, and thereby the difference between the groups is relatively small.

These findings demonstrate that circulating serum metabolites in childhood are affected by maternal dietary exposure as early as the fetal period. We observed beneficial effects in the infants, such as an increase in concentrations of n–3 fatty acids and lipids in HDL and LDL particles due to maternal supplementation of fish oil alone or fish oil in combination with probiotics cross-sectionally at the age of 6 mo; the effect of the supplementation persisted from 1 y to 5–6 y of age. This is evidence that the offspring’s metabolic profile can be modulated through the mother’s diet, which may also, at least partially, explain the clinical findings shown previously [14,15]. These findings suggest that a dietary intervention with fish oil may benefit the metabolism of children born to mothers with overweight and obesity. However, further research will be needed to translate these interesting observations on metabolomics into clinically meaningful outcomes in children.

## Author contributions

KL, VH designed the research; KL, NH, and JV conducted the research; DM analyzed data; LL and TV supervised the analysis; VH, DM, KL, NH, and CZ interpreted results; VH drafted the manuscript; VH, NH, LS, DM, and KL wrote the article; KL was responsible for supervision and project administration. All authors reviewed and commented on the article. KL had primary responsibility for final content and all authors: read and approved the final manuscript.

## Data availability

The datasets are not available due to the fact that they contain information that could compromise the privacy and consent of the participants.

## Declaration of Generative AI and AI-assisted technologies in the writing process

None.

## Funding

The work was supported by the Academy of Finland (#258606), State research funding for university-level health research of the Turku University Hospital Expert Responsibility Area, Sigrid Juselius Foundation, the Diabetes Research Foundation, the Juho Vainio Foundation, Business Finland (#3486/31/2015), and The Finnish Cultural Foundation. DM’s contribution to this work has been co-funded by European Union’s Horizon Europe Framework program for research and innovation 2021-2027 under the Marie Skłodowska-Curie grant agreement No 101126611. These funding bodies have no role in the design of the study, collection and analysis of data and decision to publish.

## Conflict of interest

The authors report no conflicts of interest.
