# Supplementary material for: The Impact of Maternal Supplementation of Fish Oil and/or Probiotics During Pregnancy on the Serum Metabolomic Profile From Infancy to Childhood: Secondary Analysis of a Randomized Placebo-Controlled Trial
Source: Curr Dev Nutr. 2025 Sep 9;9(10):107553. doi: 10.1016/j.cdnut.2025.107553 (PMC12538415; doi:10.1016/j.cdnut.2025.107553)
Supplement: multimedia component 2 [file mmc2.docx]

**SUPPLEMENTARY MATERIAL** to Current Developments in Nutrition

**FIGURES**

**The impact of maternal supplementation of fish oil and/or probiotics during pregnancy on the serum metabolomic profile from infancy to childhood: secondary analysis of a randomized placebo-controlled trial**

Houttu V et al.


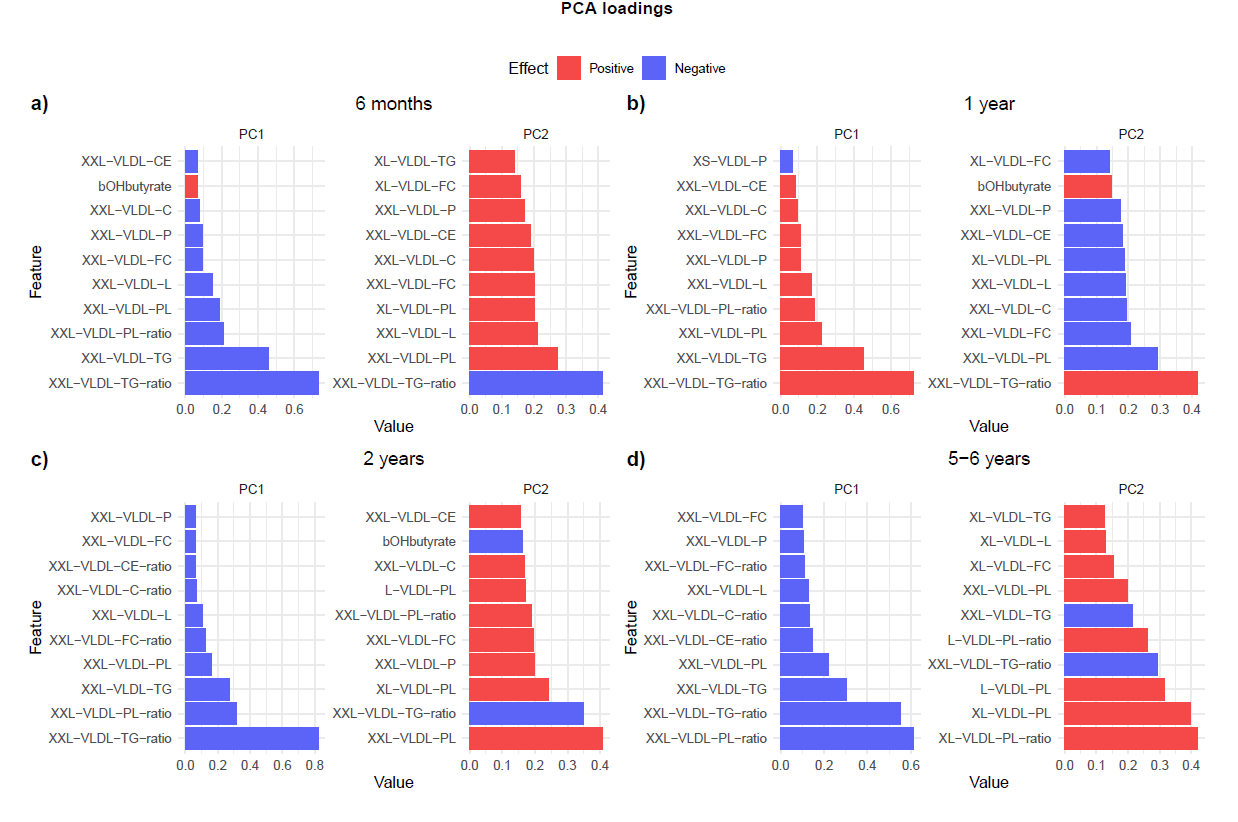


**Supplementary Figure 1.** Loadings of the Principal Components (PC) in the Principal Component Analysis (PCA) for the metabolite profiles at 6 months of age, 1 year of age, 2 years of age, 5–6 years of age. Cholesterol C; cholesteryl esters, CE; large, L; medium, phospholipids, L; small, triglycerides, TG, very large, XL; extremely large, XXL.


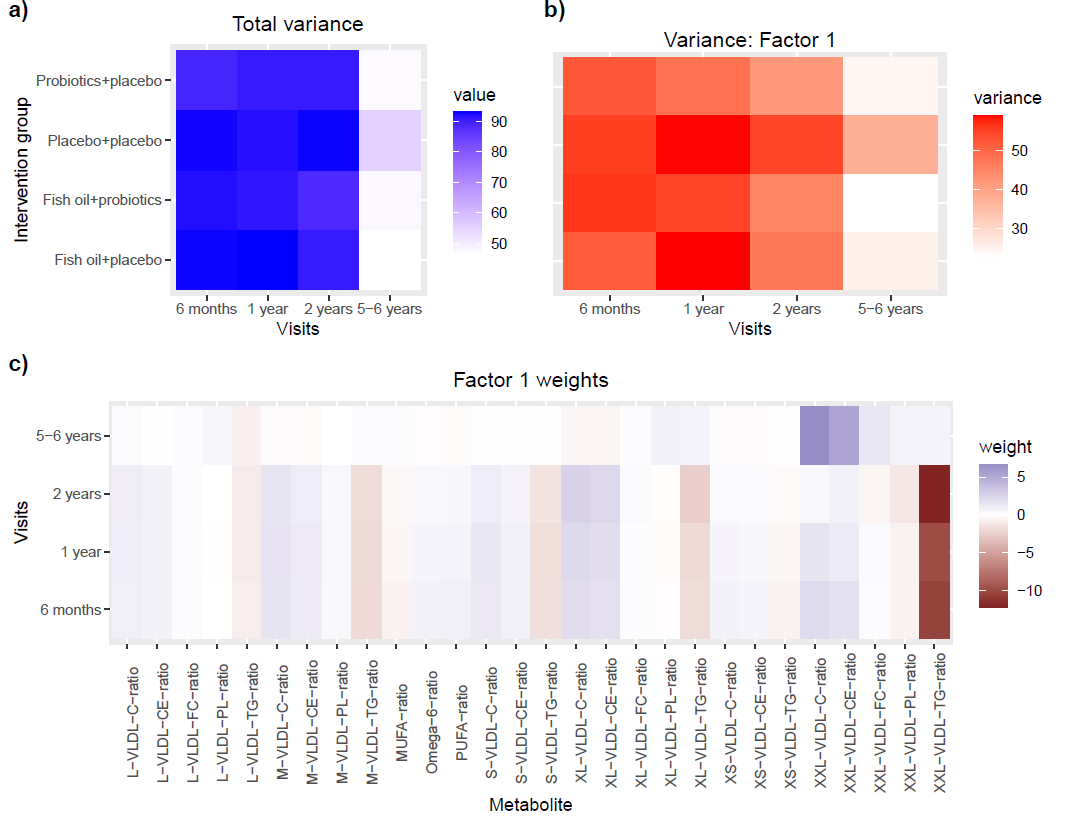
**Supplementary Figure 2.** MOFA2 multi-group framework analysis explaining the variability (a) in each intervention group at 6 months of age, 1 year of age, 2 years of age and 5–6 years of age. The figure shows the total explained variance of ratios of metabolites (a), the explained variance of ratios of metabolites in Factor 1 (b) and the weights of the associated top 10 ratios of metabolites per visit explaining variability in Factor 1 (c). Cholesterol, C; cholesteryl esters, CE; large, L; medium, M; phospholipids, PL; small, S; triglycerides, TG, very large, XL; extremely large, XXL, very small, XS


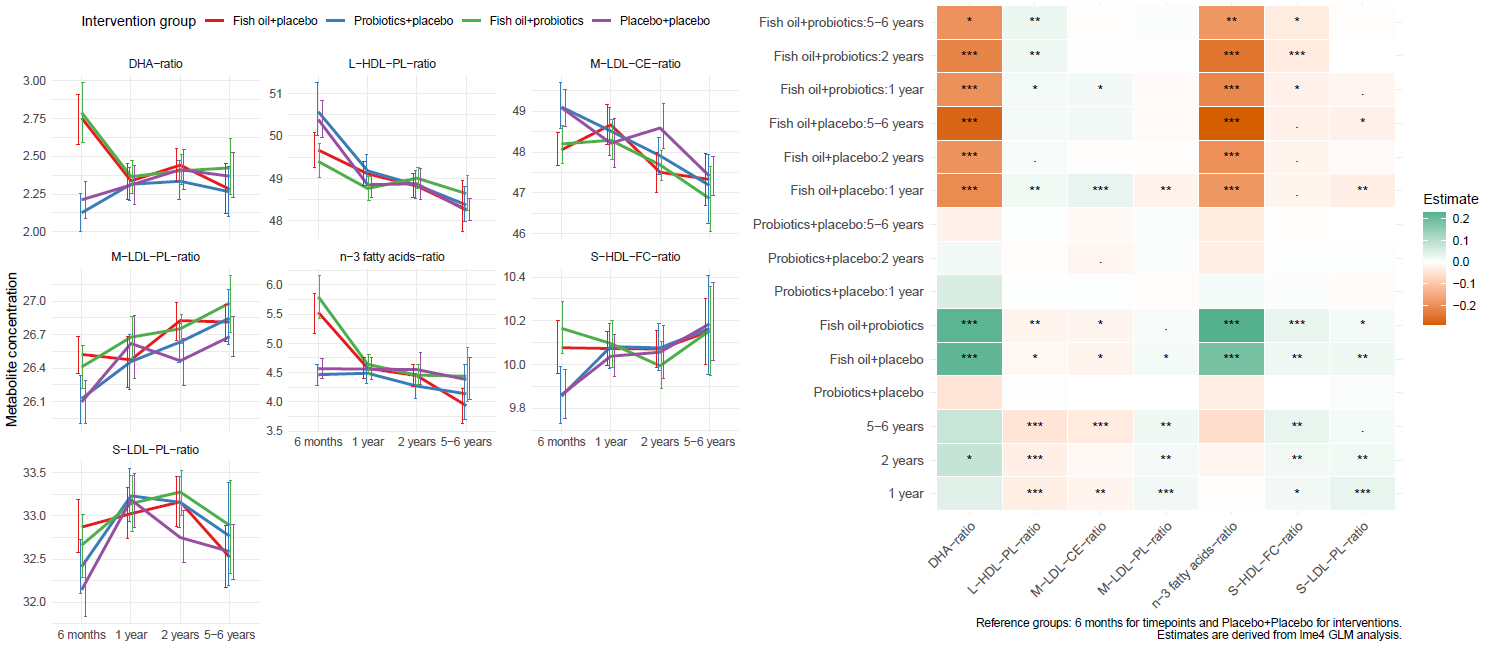
**Supplementary Figure 3.** Interaction between the maternal intervention and time with respect to the ratios of serum metabolites of children. Only the metabolites that differed statistically significantly between the intervention groups at 6 months were chosen for this analysis. Linear regression model adjusted for child sex, maternal BMI and smoking. Cholesteryl esters, CE; DHA, docosahexaenoic acid; free cholesterol, FC; large, L; medium, M; phospholipids, small, S
